# Water Availability, Soil Characteristics, and Confounding Effects on the Patterns of Biocrust Diversity in the Desert Regions of Northern China

**DOI:** 10.3389/fpls.2022.835668

**Published:** 2022-05-26

**Authors:** Jingyao Sun, Xinrong Li

**Affiliations:** ^1^Shapotou Desert Research and Experiment Station, Northwest Institute of Eco-Environment and Resources, Chinese Academy of Sciences, Lanzhou, China; ^2^Gansu Provincial Key Laboratory of Stress Eco-Physiology in Cold and Arid Regions, Lanzhou, China

**Keywords:** biocrusts, richness, desert region, confounding effect, canonical ordination, structural equation model

## Abstract

The species diversity of biocrusts is an important community characteristic in determining their multiple ecosystem functions. Hence, understanding the diversity patterns of biocrusts and their environmental drivers is of fundamental importance. However, explain variables often correlated with each other; thus, the confounding effects among them may arise and result in spurious causal relationships and biased ecological inferences. In this study, we investigated the richness of three biocrust-forming components (mosses, lichens, and cyanobacteria–algae) and their environmental variables across six desert regions of northern China. A comparison between conventional redundancy analysis (RDA) and structural equation model (SEM) was conducted to study the environmental driver-richness relationship and the confounding effects. Our results showed that three latent variables related to water availability, soil texture, and soil salinity and sodicity, could account for the main environmental variations and explain the diversity patterns of biocrusts at the intracontinental scale. Water availability was positively and negatively related to the richness of mosses and cyanobacteria–algae, respectively, while soil texture was positively related to the richness of lichens. In addition, environmental variables confounded with each other caused distinct driver-richness relationships between results of RDA and SEM. Therefore, we suggest that future multivariable studies should utilize path analysis in conjunction with conventional canonical ordination to facilitate more rigorous ecological inferences.

## 1. Introduction

The community of biological soil crusts (biocrusts), which include various species of free-living cyanobacteria, algae, lichens, mosses, and other microorganisms, is an important part of arid ecosystems (Li et al., 2003; Bowker and Belnap, 2009; Bowker et al., 2014). The distribution and development of biocrusts are important indicators of the status of dryland ecosystems (Chen et al., 2020). The species diversity of biocrusts is also a determining factor influencing the ecosystem functions of biocrusts, such as soil stabilization, nitrogen fixation, carbon sequestration, and hydrological regulation (Chaudhary et al., 2009; Belnap, 2010; Castillo-Monroy et al., 2010; Bowker et al., 2013; Li et al., 2013). Therefore, understanding the diversity patterns of biocrusts and their environmental drivers is of fundamental importance.

At the intracontinental scale, multiple factors such as the climatic regime and edaphic conditions, determine the diversity of biocrusts (Eldridge and Tozer, 1997a; Rivera-Aguilar et al., 2006; Zedda and Rambold, 2009). Among these parameters, the latitude and altitude are the primary factors shaping the thermal regime, while continentality and rain shadows further shape the precipitation regime, which determines the basic type and distribution of biocrusts in dryland ecosystems (Zedda and Rambold, 2009; Root and Mccune, 2012; Li et al., 2017). Parent materials and the degree of soil development also vary geographically, which determines the physical and chemical properties of soil, commonly including the texture, salinity, sodicity, and fertility of the soil (Büdel et al., 2009; Ochoa-Hueso et al., 2011; Belnap et al., 2014; Steven et al., 2014). Furthermore, variations in the topsoil texture often involve a change in the dominant functional group among biocrust-forming cyanobacteria, algae, lichens, and mosses, with cyanobacteria and algae dominating coarse-textured soil and lichens and mosses dominating fine-textured soil (Eldridge et al., 2006). In arid regions, switching from gypsic calcareous substrates to non-gypsic calcareous substrates is associated with prominent gradients of soil salinity and sodicity, which also lead to the significant species turnover of biocrusts (Büdel et al., 2009; Ochoa-Hueso et al., 2011). In contrast, fertility is regarded as a less important soil property for the distribution and diversity of biocrusts because the slow growth rate in the arid region makes them quite unlikely to be nutrient limited (Li et al., 2010; Weber et al., 2016).

However, in empirical studies, these variables are often correlated with each other, making it difficult for researchers to discover actual causations and even leading to fraudulent cause-effect relationships. According to the definition of confounding effect, the detected correlation between variables *X* and *Y* cannot reflect their causality or intrinsic relationship because this observed correlation often comes from an undetected confounding variable *Z*, which covaries with both *X* and *Y* (Shipley, 2016; Vellend, 2016). Unfortunately, previous research on the diversity pattern of biocrusts largely overlooked the role of confounding variables (Eldridge et al., 2006; Ochoa-Hueso et al., 2011; Root and Mccune, 2012). Therefore, we wondered whether some observed influences of environmental variables on the diversity of biocrusts are confounded by other variables, thus leading to exaggerated roles.

To address this knowledge need, we investigated the composition and diversity of biocrust communities and the multiple corresponding environmental variables at the intracontinental scale. Samples were collected from 6 biocrust typical habitats along the main east–west precipitation gradient among the main desert regions of northern China. We used a structural equation model (SEM) to disentangle the confounding influences among these drivers because SEMs provide good solutions to eliminate confounding effects, as they evaluate the “partial” influence from *X* to *Y* that excludes the indirect influence of *Z*→*X*→*Y* (Lefcheck, 2016; Shipley, 2016). That is, the evaluated effect *X*→*Y* reflects their direct relation without the interference of *Z*. In addition, conventional redundancy analysis (RDA) was also conducted to compare the findings with the SEM results in order to reveal the confounding effects. We aimed to clarify the main environmental drivers for the variations in biocrust community diversity and biomass across the desert regions of northern China, which provides a benchmark for studies focusing on specific drivers at smaller scales. This study can help us build more accurate estimates of the relationships between environmental drivers and biocrust diversity than their apparent bivariate relationships.

## 2. Materials and Methods

### 2.1. Study Sites

**Figure 1:**
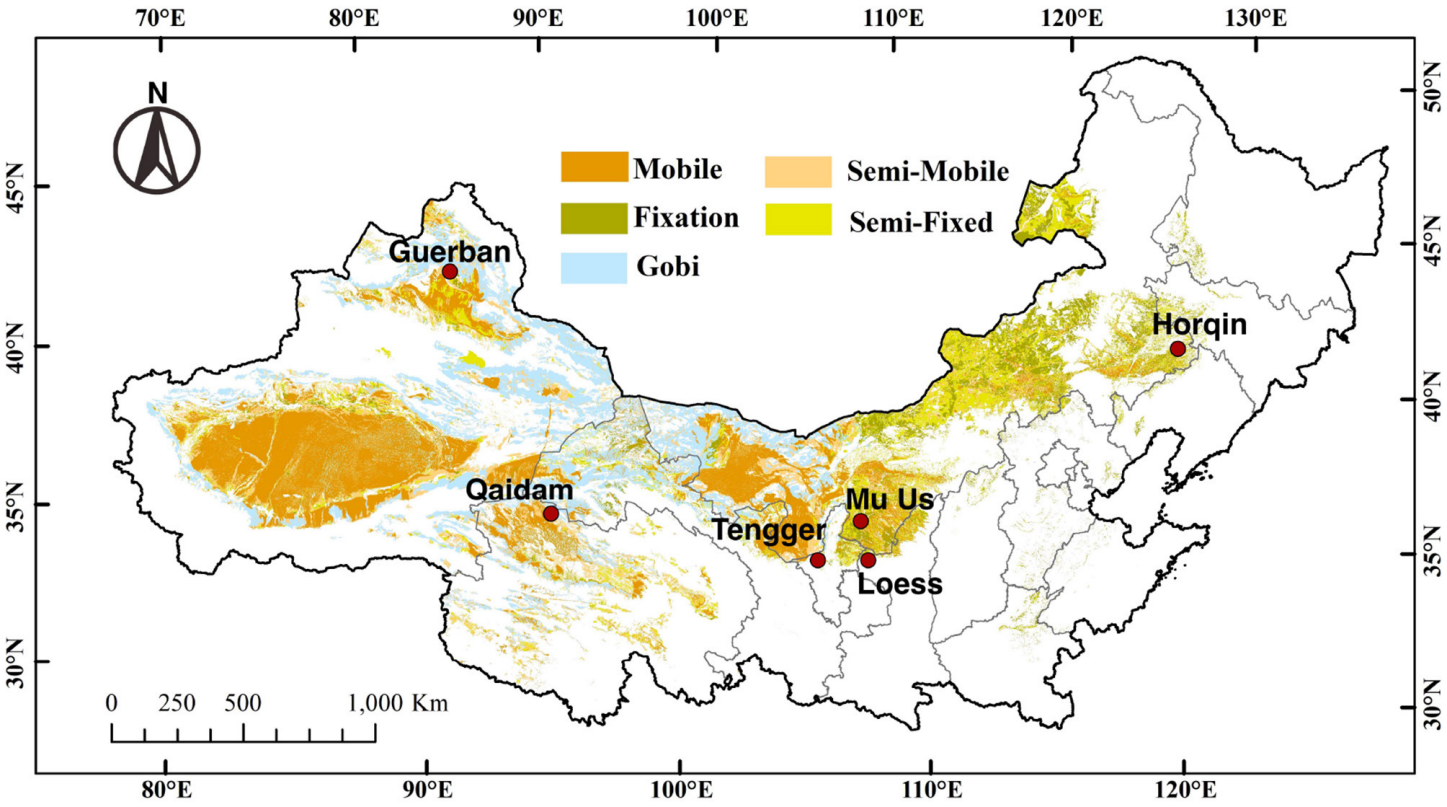
The distribution of 6 study sites dispersed across different desert regions of northern China, i.e., the Horqin Desert (Horqin), western Loess Plateau (Loess), Mu Us Desert (Mu Us), Tengger-Alxa Desert (Tengger), Guerbantunggut Desert (Guerban), and Qaidam Desert (Qaidam) (Yan and Wang, 2019). The different colors mark the different classifications of deserts.

This study was conducted at 6 sites dispersed across northern China, i.e., the Horqin Desert (Horqin), western Loess Plateau (Loess), Mu Us Desert (Mu Us), Tengger-Alxa Desert (Tengger), Guerbantunggut Desert (Guerban), and Qaidam Desert (Qaidam) (Figure 1). All sites are longitudinally arranged from the east (offshore) to west (inland) with a precipitation gradient ranging from 80 mm to 450 mm and an opposite gradient for annual evaporation (from 2,900 to 2,100 mm). The mean annual temperature ranges from 4.6 to 12.3°*C*. Among the 6 sites, the regional climatic regime and vegetation composition are distinct, and the details are listed in Supplementary Table S1 (Horqin: Zhao et al., 2010a; Loess: Zhao et al., 2010b; Mu Us: Guan and Cao, 2019; Tengger: Li et al., 2003; Guerbantunggut: Zhang et al., 2002; Qaidam: Zhang et al., 2019).

### 2.2. Biotic Investigation and Identification

Biotic investigations were conducted in September 2014. In the study sites of Horqin, Loess, Mu Us, Tengger, Guerban, and Qaidam, respectively, 60, 40, 60, 40, 60, and 20 plots with a size of 10 × 10*m* were set. We randomly scheduled the location of plots on the map of the six deserts, with the typical vegetation and biocrust types under the regional climatic conditions without human disturbance. Then, we found the locations in the field using GPS (Garmin, Taiwan, China) and selected a new location 100–1,000 m away from a locally typical place when the predetermined location was disturbed. At each location, we chose flat terrain to place the plot in order to avoid topographic interference.

For the vegetation survey, a 5 × 5*m* quadrat was placed in the center of each plot. We identified the perennial plants and recorded their cover (cvP) for each quadrat using the classifications of Su et al. (2011). A similar survey for annual plants was excluded because the cover of annual plants is more related to impulsive precipitation events in drylands. For the biocrust survey, a 30 × 30*cm* quadrat was randomly nested in the 5 × 5*m* quadrat and at interspace among shrubs. This small quadrat was partitioned into 144 equal-sized 2.5 × 2.5*cm* cells by nylon wire, and the biocrust layer in the small quadrat was sprayed with distilled water to make the biocrust components more visually recognizable. Then, we recorded the cover for three visible biocrust components (cyanobacteria-algae, lichens, and mosses) by measuring how many of the 144 cells each component occupied (Magurran, 1988). The richness of lichen (RLi) and moss (RMo) species was investigated in the field according to the classification of Li et al. (2016), and some specimens were taken to the laboratory to confirm their identity. The fresh cyanobacteria-algae crusts (3–5 mm thick at the surface) were also collected by ring knife or sterile shovel. The diacritical morphological traits, including cell shape for both intercalary and end cells, width and length of intercalary cells, presence or absence of constriction at the cross wall of necridic cells and of a sheath, the color of the sheath, number of trichomes per filament, presence or absence of heterocysts, width and length of heterocysts were identified *via* microscopy to count the richness of cyanobacteria-algae (RCA) at the genus level (Castenholz, 2001; Taton et al., 2003). The total biomass (BM) was estimated by the total content of chlorophyll a and b extracted from 0.5 g fresh weight of mixed biocrust specimen (without soil). The specimen were powdered with liquid nitrogen and pigments were extracted with 80% acetone until complete bleaching (Lichtenthaler and Wellburn, 1985).

### 2.3. Abiotic Sampling and Measurement

After completing the biocrust survey in the field, we took a sample of topsoil (0–5 cm under the biocrust layer) from the biocrust quadrat to evaluate the soil conditions. Then, the soil samples were air-dried at ambient temperature to a constant weight (3–5 days), crushed, and sieved with a 2-mm mesh for subsequent measurements. The soil particle sizes were analyzed using the pipette method, including the silt content (Silt, 0.002–0.05 mm) and clay content (Clay < 0.002 mm) (Hopmans and Bristow, 2001). The soil pH was analyzed in a 1:5 soil-water suspension *via* a portable multimeter (HQ30d, Hach Company, USA). The soil soluble salt (SS) was determined by the dry weight method of soil extracting solution (Nanjing Institute of Soil Research, 1980). The *CaCO*_3_ accumulation (Ca) was determined by volumetric analysis of the carbon dioxide (Nanjing Institute of Soil Research, 1980). The soil organic matter (SOM) was determined with the dichromate oxidation method described by Nelson and Sommers (1982). The total nitrogen content (TN) was measured *via* elemental analysis (vario MACRO cube, Elementar Analysensysteme, Germany), while the total phosphorus content (TP) was measured *via* Mo-Sb colorimetry (Nanjing Institute of Soil Research, 1980), and the total potassium content (TK) was measured by the method described by Nelson and Sommers (1982). In addition, the topsoil water content (TSW) was measured *via* the oven-drying method and transformed into the volumetric moisture content using the bulk density of the soil (Nanjing Institute of Soil Research, 1980). The mean soil water content (SWC) was calculated from monthly (May-September) topsoil samplings (0–10 cm) that were collected with ring knives outside the quadrats and inside the plots to avoid interference with other samplings, and at least 1 week after a rain event to lower contingency.

### 2.4. Structural Equation Modeling

**Figure 2:**
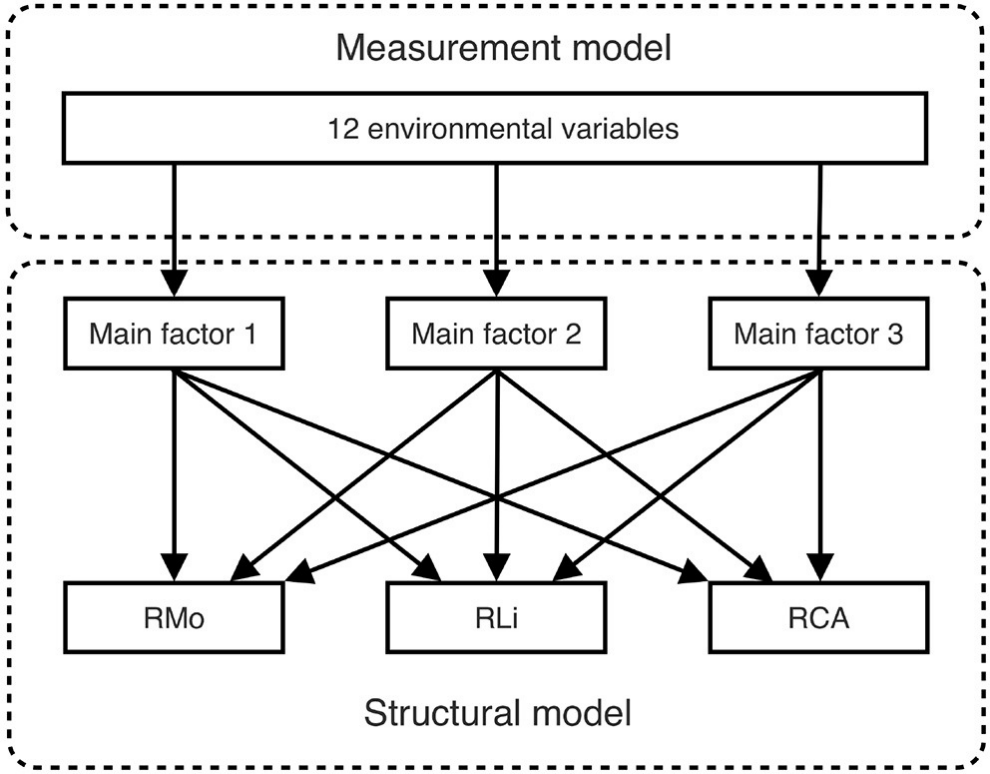
Measurement and structural parts of the structural equation model (SEM). The measurement model condenses interrelated environmental variables into a few latent variables, whereas the structural model analyzes the influences of these latent variables on the richness of the three biocrust-forming components (mosses, lichens, and cyanobacteria-algae).

The SEM with latent variables consists of both a measurement model and a structural model, as illustrated in Figure 2. The measurement model can eliminate redundancy among 12 environmental variables by mapping them to several latent variables. First, we decided how many main latent variables are representative of the observed variables; specifically, the Kaiser criterion, Cattell's scree test, and parallel analysis were implemented (Kaiser, 1960; Cattell and Jaspers, 1967; Hayton et al., 2004). Second, we applied different cluster methods (Ward.D, Ward.D2, single-linkage, complete-linkage, average-linkage, and McQuitty) to preliminarily divide the environmental variables into the main factors (Legendre and Legendre, 2012). In contrast, the structural model is quite simple: it links the latent variables to the richness of each of the three biocrust-forming components (mosses, lichens, and cyanobacteria-algae). The SEM goodness-of-fit evaluates both the measurement model and the structural model, but our measurement model is of excessive redundancy that the SEM fitting will inevitably fail. Therefore, we conducted a stepwise deletion of observed variables to latent variables until the SEM fit the data. We evaluated the overall goodness-of-fit of the SEM by applying a χ^2^ test, a root mean square error of approximation (RMSEA) test, the comparative fit index (CFI), and the Tucker-Lewis index (TLI) (Barrett, 2007; Beaujean, 2014). After the observed variables were deleted, we obtained the final model, which could (without redundancy from correlated environmental variables) concisely reflect the total influences on the diversity pattern of biocrusts. Notably, the interference from one variable to another could be disentangled to help researchers evaluate the real important variables.

### 2.5. Statistical Analyses

The difference in abiotic and biotic indicators between study sites was analyzed by one-way analysis of variance (ANOVA) and multiple comparisons of least significant difference (LSD) method at *P* < 0.01. To illustrate the correlations between the 12 environmental variables and 3 richness indexes, we evaluated their Pearson correlation coefficients and constructed a clustering tree by Ward.D2 method to show the relationships among the environmental variables. We also employed the commonly used redundancy analysis (RDA) method to analyze the relationships between the environmental variables and response variables (i.e., RMo, RLi, and RCA in this study). We aimed to compare the results between the RDA and SEM to show the confounding influences between variables. All data analyses were performed in the R environment (version 3.5.2). The cluster analysis and RDA were performed with the “vegan” package (Simpson et al., 2010), and the SEM analysis was performed with the “lavaan” package (Rosseel, 2012).

## 3. Results

The change in the proportion of fine particles (Silt and Clay) was inconsistent with the longitudinal gradient from east to west (Table 1). The highest Silt and Clay reached at the western Loess Plateau with values of 61.23(6.5)% and 13.3(1.29)%, respectively. The lowest Silt and Clay reached at Mu Us Desert with values of 0.45(0.26)% and 0.51(0.1)%, respectively. The soil pH at all study sites was alkaline (7.46–8.73). The SS and Ca were highest at Qaidam Desert (1.71(0.37) *gkg*^−1^ and 7.7(0.62) *gkg*^−1^, respectively). The SOM, TN, TP, and TK were all significantly different among all sites (*P* < 0.01). The TSW significantly decreased (*P* < 0.01) at precipitation gradient from east to west (from 450 to 80 mm, Appendix A).

**Table 1:** Abiotic indicators (mean (SD)) of topsoil under the biocrust layer at six sites in northern Chinese deserts.

**Study sites**	**Silt**	**Clay**	**pH**	**SS**	**Ca**	**SOM**	**TN**	**TP**	**TK**	**TSW**
**(Sampling number)**	**(%)**	**(%)**		**(gkg^−1^)**	**(gkg^−1^)**	**(gkg^−1^)**	**(gkg^−1^)**	**(gkg^−1^)**	**(gkg^−1^)**	**(%)**
Horqin (60)	7.27(5.22)d	0.68(0.42)d	7.46(0.14)e	0.23(0.07)d	0.35(0.1)c	7.58(4.63)b	0.8(0.39)a	0.18(0.0)3d	2.44(0.38)a	14.27(1.27)a
Western Loess Plateau (40)	61.23(6.5)a	13.3(1.29)a	8.54(0.18)ab	1.43(0.13)b	0.97(0.1)b	5.26(0.08)c	0.58(0.08)b	1.02(0.08)a	1.88(0.18)b	9.78(0.66)b
Mu Us Desert (60)	0.45(0.26)e	0.51(0.1)d	7.62(0.41)d	0.28(0.08)d	0.32(0.07)c	11.41(4.86)a	0.8(0.3)a	0.4(0.14)b	1.43(0.42)c	7.12(2.31)c
Tengger-Alxa Desert (40)	22.14(2.82)b	2.24(0.94)b	7.84(0.14)c	0.88(0.22)c	0.21(0.03)d	7.07(0.37)bc	0.45(0.05)b	0.34(0.04)c	1.96(0.13)b	2.02(0.74)d
Guerbantunggut Desert (60)	16.64(3.64)c	1.85(0.22)c	8.48(0.51)b	0.29(0.08)d	0.95(0.16)b	2.38(0.62)d	0.5(0.66)b	0.38(0.09)bc	1.99(0.35)b	2.02(0.54)d
Qaidam Desert (20)	2.12(0.33)e	2.49(0.25)b	8.73(0.32)a	1.71(0.37)a	7.7(0.62)a	0.38(0.07)d	0.37(0.1)b	0.19(0.0)5d	2.08(0.25)b	2.17(0.26)d

*The lowercase letters denote the significant difference at P < 0.01*.

**Figure 3:**
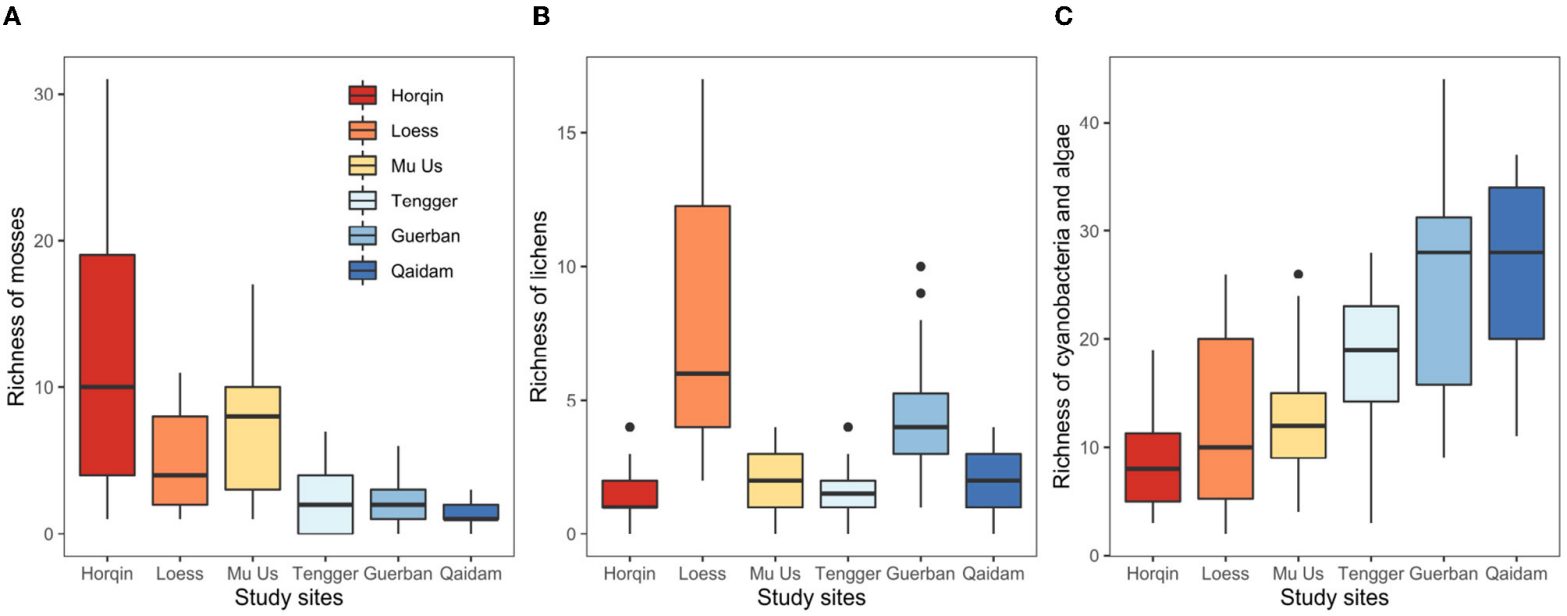
Biotic indicators include the richness of mosses **(A)**, lichens **(B)**, and cyanobacteria-algae **(C)** the biomass of biocrusts and the cover of perennial plants of the study sites (mean (SD)) in northern Chinese deserts.

The richness of mosses and cyanobacteria–algae changed across the six study sites along either a longitudinal gradient or a precipitation gradient (Appendix A). From the east (Horqin) to the west (Qaidam) (Figures 3A,C), the richness of mosses decreased from 11.98(8.53) to 1.29(0.78), respectively. The richness of cyanobacteria–algae increased from 8.87(4.28) to 25.57(8.76) (Table 2). The richness of lichens did not show any regularity with the longitudinal gradient (Figure 3B). The BM and cvP also showed decreased trend from east to west except for the point of BM at Qaidam Desert (Table 2).

**Table 2:** Biotic indicators include the richness of mosses, lichens, and cyanobacteria-algae, the biomass of biocrusts, and the cover of perennial plants of the study sites (mean (SD)) in northern Chinese deserts.

**Study sites**	**Richness**			**BM**	**cvP**

**(Sampling number)**	**Mosses**	**Lichens**	**Cyanobacteria-algae**	**(mgcm^−2^)**	**(** **%** **)**
Horqin (60)	11.98(8.53)a	1.5(1.11)c	8.87(4.28)d	5.83(1.88)ab	43.65(11.7)a
Western Loess Plateau (40)	5(3.22)bc	7.72(4.79)a	11.95(8)cd	5.33(2.38)b	40.8(8.96)a
Mu Us Desert (60)	7.38(4.12)b	1.95(1.16)c	12.42(4.67)c	4.74(2.32)b	38.17(17.45)ab
Tengger-Alxa Desert (40)	2.38(2.14)cd	1.65(1.14)c	17.62(8.01)b	3.17(1.85)c	32.33(6.8)bc
Guerbantunggut Desert (60)	2.03(1.28)d	4.23(2.1)b	24.63(8.78)a	1.97(0.82)c	27.53(11.55)cd
Qaidam Desert (20)	1.29(0.78)d	2.14(1.2)c	25.57(8.76)a	7.2(5.61)a	21.62(9.2)d

*The lowercase letters denote the significant difference at P < 0.01*.

**Figure 4:**
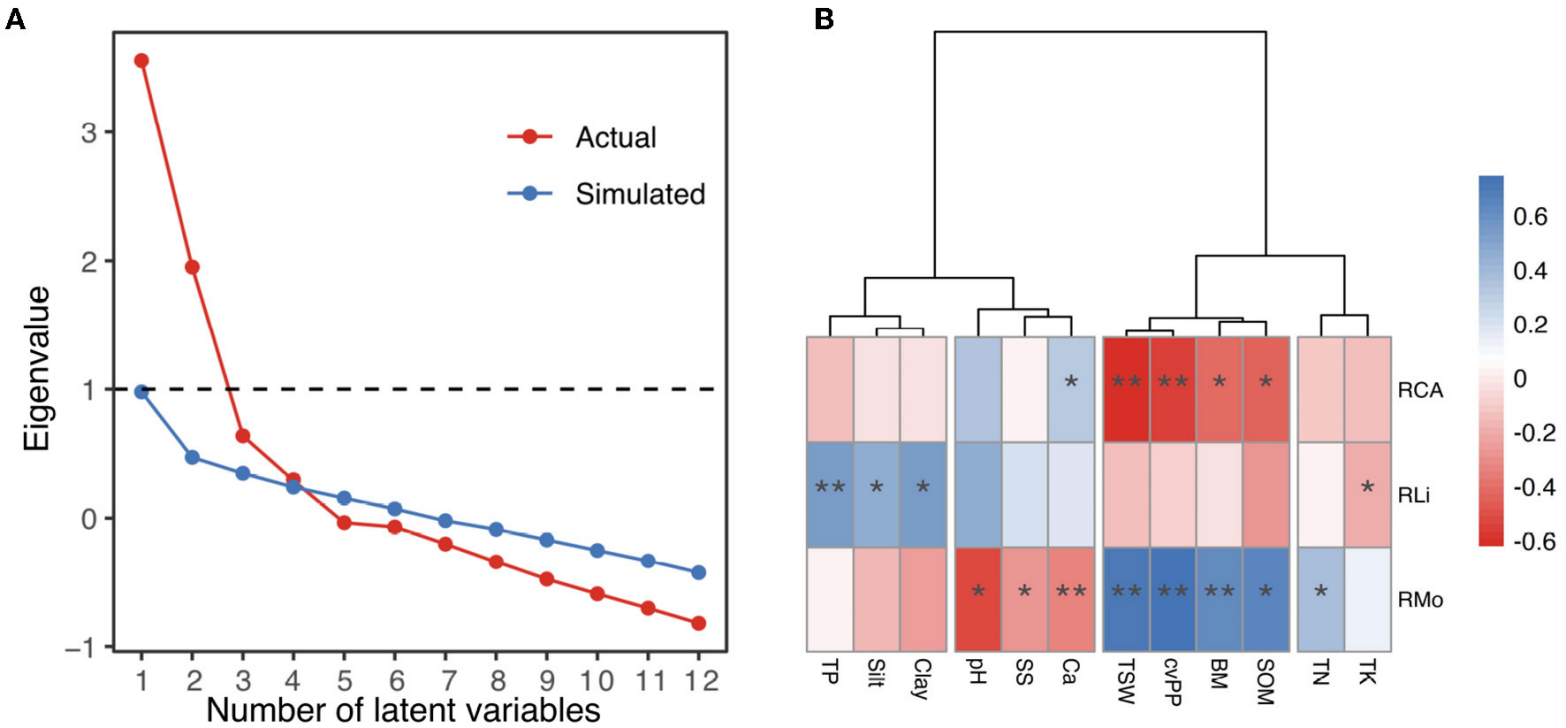
**(A)** Scree plot of the successive eigenvalues to determine the number of representative latent variables of environmental variables. The horizontal dashed line marks two eigenvalues greater than 1 (Kaiser criterion). The break point occurs between 3-5 at the rank of actual eigenvalues (Cattell's scree test). Four eigenvalues from the actual data correlation matrix are higher than eigenvalues from the random correlation matrices (parallel analysis). **(B)** Correlations between the environmental variables and richness of mosses, lichens, and cyanobacteria-algae. The cluster tree divides the environmental variables into four groups according to the scree plot. Significant code: **P < 0.01 and *P < 0.05.

Figure 4A shows the results of the three methods used to determine the number of latent variables. According to the Kaiser criterion, which retains variables with eigenvalues greater than 1, 2 components are appropriate for our data (Kaiser, 1960). Cattell's scree test aims to find the break point within the rank of eigenvalues because few major variables account for most of the variance and numerous minor variables account for the relatively consistent variance, namely, the steep “cliff” and flat “scree” on the plot, respectively (Cattell and Jaspers, 1967). The break point occurs at either 3 to 5 in Figure 4A, indicating that 3 to 5 latent variables are representative of the total variance. The parallel analysis indicates that 4 latent variables are appropriate because the nontrivial variables from the actual data with a valid underlying variables structure should have eigenvalues larger than the parallel variables derived from random data (Hayton et al., 2004).

Thus, the cluster method of the ward.D, ward.D2, complete linkage, average-linkage, and McQuitty suggested clustering the environmental variables into 4 groups: Clay, Silt, and TP; Ca, SS, and pH; SOM, BM, cvP, and TSW; and TK and TN (Appendix B). As shown in ward.D2 cluster tree (Figure 4B), the environmental variables within clusters exhibited similar correlations with the richness indexes, whereas those between the clusters displayed different correlations. Clay, Silt, and TP were moderately positively correlated with RLi (*r* = 0.54, 0.45, and 0.54, respectively), whereas Ca, SS, and pH were moderately negatively correlated with RMo (*r* = –0.34, –0.29, and –0.51, respectively). SOM, BM, cvP, and TSW were strongly positively correlated with RMo (*r* = 0.66, 0.62, 0.74, and 0.72, respectively) but negatively correlated with RCA (*r* = -0.44, –0.42, –0.55, and –0.62, respectively). However, TK and TN had very weak relationships with each richness variable; thus, we excluded these variables from subsequent analyses.

The stepwise deletion of observed variables began with the remaining three clusters of environmental variables, and the measurement paths with the smallest standardized path coefficient (SPC) were deleted until the SEM fit the data (Table 3). The SEM in step 1 had a poor fit to the data according to the χ^2^ test (χ^2^ = 351.733, df = 16, *P* < 0.001), and the RMSEA, TLI, and CFI were 0.14, 0.888, and 0.973, respectively. In contrast, the SEM in step 3 fit the data with χ^2^ = 19.221, df = 54, *P* = 0.038, and the RMSEA, TLI, and CFI were 0.063, 0.982, and 0.992, respectively. Despite the *P*-value slightly failing the chi-squared criteria of 0.05 level, we consider our model to be a good fit according to other indicators.

**Table 3:** Results of the structural equation model (SEM) fitting and stepwise deletion of observed variables.

**Step**	**Latent variables**	**Observed variables**	**χ^2^**	**df**	***P*-value**	**RMSEA**	**TLI**	**CFI**
Step 1	MF1	TSW+cvP+SOM+BM	351.733	54	<0.001	0.140	0.888	0.923
	MF2	Clay+Silt+TP						
	MF3	pH+Ca+SS						
Step 2	MF1	TSW+cvP+SOM	351.733	54	<0.001	0.106	0.949	0.973
	MF2	Clay+Silt						
	MF3	pH+Ca						
Step 3	MF1	TSW+cvP	19.221	16	0.038	0.063	0.982	0.992
	MF2	Clay+Silt						
	MF3	pH+Ca						

**Figure 5:**
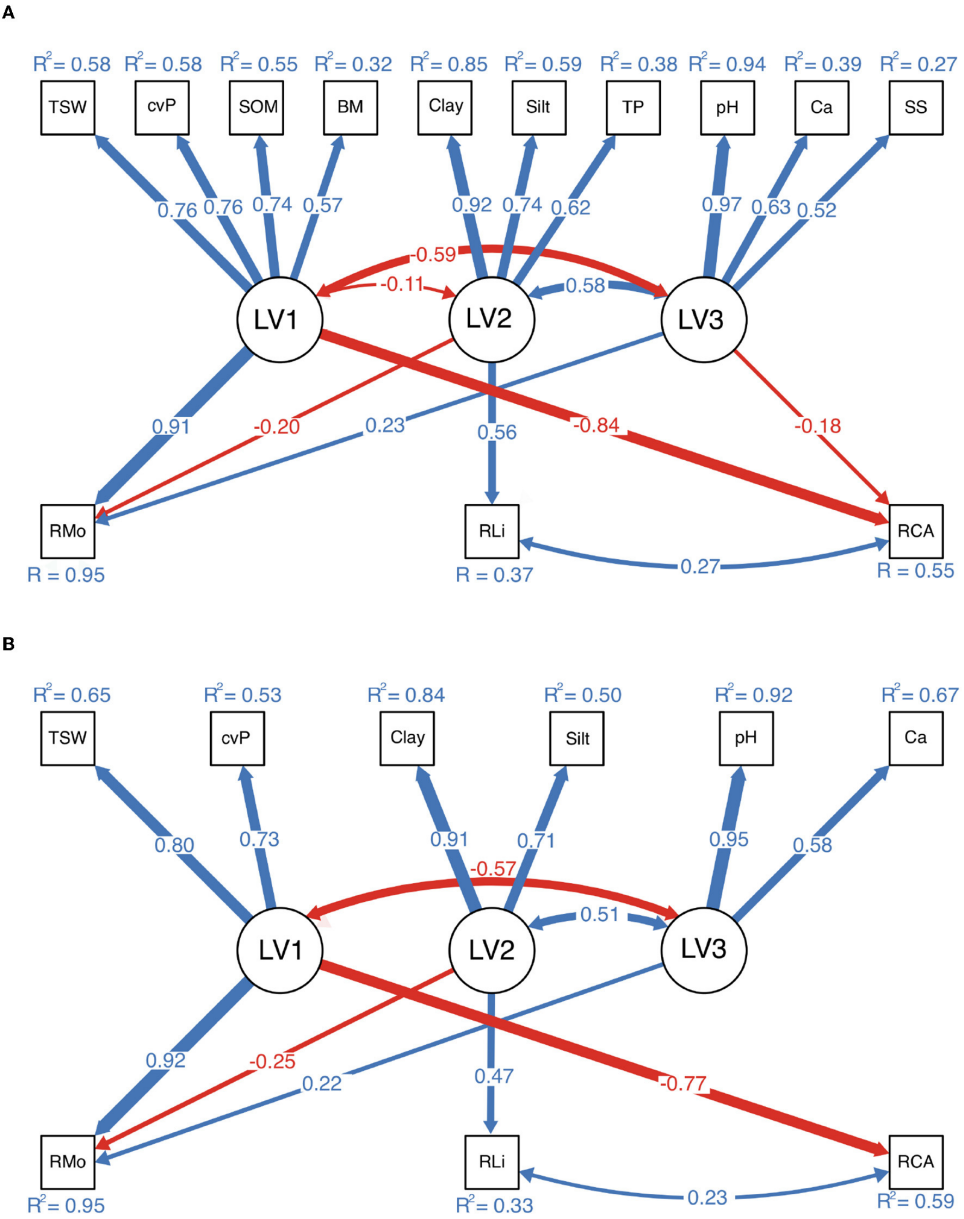
Results of structural equation modeling. **(A)** Measurement model with redundant observed variables. **(B)** Model after the deletion of observed variables. The two models have very similar structures and standardized path coefficients (SPCs) in the structural parts. Only the paths with a significant level of 0.05 or lower are plotted. The thickness of each arrow is proportional to the SPC labeled on the corresponding path, with blue and red reflecting positive and negative values, respectively.

The structure and SPCs depicted in Figures 5A,B are basically the same. RMo was strongly influenced by MF1 (SPC = 0.91, 0.92) and weakly influenced by MF2 (SPC = –0.20, –0.25) and MF3 (SPC = 0.23, 0.22), whereas RLi was influenced by MF2 (SPC = 0.56, 0.47), and RCA was negatively influenced by MF1 (SPC = –0.84, –0.77).

**Figure 6:**
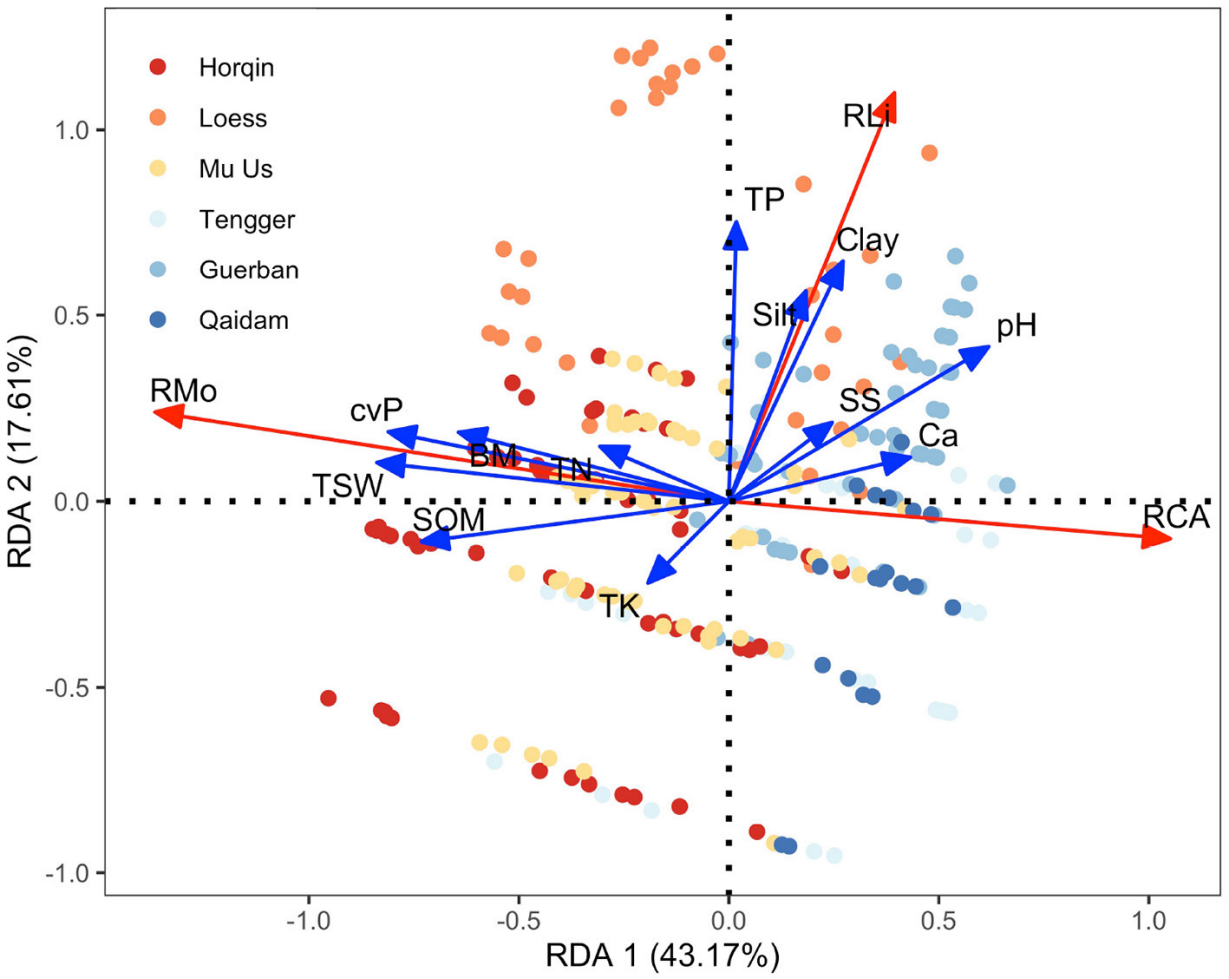
Redundancy analysis (RDA) ordination triplot showing the relationships between the environmental variables (TK, TP, SOM, BM, cvP, TSW, CA, SS, pH, Clay, Silt, and TP, marked with blue arrows) and the variations in the species richness of biocrust components (mosses, lichens, and cyanobacteria-algae, marked with red arrows). Different points indicate the samples obtained from the six study sites across the desert regions of northern China.

From the RDA results (Figure 6), RMo was positively correlated with TSW, cvP, SOM, and BM and negatively correlated with Ca and pH. RLi was positively correlated with TP, Silt, Clay, SS, and pH and negatively correlated with TK. RCA was positively correlated with Ca and negatively correlated with TSW, cvP, BM, SOM, TN, and TP. We evaluated the above correlations between the variables based on their scores on RDA axes; the results are listed in Appendix A.

## 4. Discussion

### 4.1. Main Environmental Variables at the Intracontinental Scale

In the desert regions of northern China, many environmental indicators are closely related (Figure 6). We found that environmental variations in the desert regions of northern China could be represented by four (unobserved) latent variables (Figure 4A). Conceptually, the variables in each group were also related; therefore, there may exist some substantial and latent variables that determine the variables we have measured. MF1, which was represented by TSW, cvP, SOM, and BM, reflected the local productivity of both vascular plants and biocrusts, to a large extent, is determined by precipitation in arid regions. We conceptually synthesized these variables into water availability because a high TSW is usually accompanied by high precipitation, which facilitates the growth of plants (cvP) and biocrusts (BM), resulting in the accumulation of SOM in soil (Ochoa-Hueso et al., 2011; Ju et al., 2021). MF2 reflected the soil texture in the desert regions and was determined largely by the parent materials and the degree of soil development. In northern China, the distribution of parent material is relatively independent of the longitudinal and precipitation gradients, e.g., the highest content of silt and clay occurs in the western Loess Plateau (Wang and Zuo, 2010), which supports the low correlation between MF1 and MF2 (Figure 5). TP is also correlated to Silt and Clay because fine particles provide large surface areas for phosphorus adsorption (Bronick and Lal, 2005). Desert soil usually features high salinity and sodicity, which were related to MF3, because the high rate of water evaporation and soil leaching increases the soil SS and *CaCO*_3_ in arid regions (Capo and Chadwick, 1999; Zhou and Li, 2013). Therefore, the correlations of MF3 with MF1 and MF2 can be interpreted as the influences of precipitation and soil parent materials on soil salinity and sodicity (Figure 5). N and K are important macronutrients that reflect soil fertility (Read et al., 2008; Baldarelli et al., 2021). However, the role of fertility is relatively limited because biocrusts are more subject to water limitations and soil stability in arid regions (Kidron and Benenson, 2014; Chiquoine et al., 2016), which may explain the weak correlation of MF4 (TN and TK) with biocrust richness (Figure 4B).

### 4.2. Relationship Between Environmental Drivers and Biocrust Diversities

Water availability (MF1) was the most influential latent variable on moss richness, followed by soil texture (MF2), salinity and sodicity (MF3) (Figure 5). Compared with the other biocrust–forming components, mosses have a more advanced and efficient hydraulic conductivity system and consume more water; thus, soil moisture plays a more vital role in mosses (Kidron et al., 2010; Michel et al., 2012). The strong positive relationship between the richness of mosses and MF1 generally supports diversity—productivity relationships at the geographic scale (Waide et al., 1999; van Ruijven and Berendse, 2005). Ameliorated water stress can provide more niches for mosses, and many physiological activities of mosses are activated by precipitation (Whitford, 2002; Belnap et al., 2004; Liu et al., 2016). A surprising finding was that the content of fine particles had a weakly negative influence on the richness of mosses (Figure 5). The finding contrasts those of previous studies, which have suggested that fine particles prolong the wetting time, facilitating moss performance (Reynolds et al., 2001; Kidron and Benenson, 2014). In addition, we also found a positive relationship between salinity and sodicity and the richness of mosses. This result supports the earlier observations that biocrusts on the calcareous substrate (with high *CaCO*_3_, pH, and electrical conductivity) contain a greater richness and abundance of bryophytes (Moore and Scott, 1979; Eldridge and Tozer, 1997b; Ponzetti and Mccune, 2001; Büdel et al., 2009).

The determining factor shaping the richness of lichens was soil texture, as has been frequently reported in previous studies (Eldridge, 1996; Ullmann and Büdel, 2001; Fischer and Subbotina, 2014). As lichens have low requirements for water and nutrients, by contrast, soil stability is more vital for lichens with very slow growth rates (Armstrong, 2010; Chavez et al., 2018). While the fine particles, easier to glue together by a metabolite of biocrusts and plants are an important matrix for soil stability in desert regions (Bowker et al., 2012). Admittedly, the variation of RLi explained by our model was only 33% and no single variable is strongly correlated to it, suggesting the potential role of the other drivers such as biogeographic species pool or environmental disturbance regime (Büdel, 2003; Ferrenberg et al., 2015). One interesting finding was the strong negative influence of water availability on the richness of cyanobacteria–algae. However, we do not know the origin of this relationship, which deserves further study in the future.

### 4.3. Confounding Influences Among the Environmental Variables

A confounding effect arises when the spurious correlation between putative cause *X* and ostensible effect *Y* is actually caused by their strong relationship with *Z*. That is, some observed bivariate correlations are actually confounded by other variables; thus, any ecological inference based on a spurious correlation is unreliable (Shipley, 2016; Vellend, 2016). By comparing results between SEM and RDA, some bivariate correlations are different or even opposite, which indicates the confounding influences among the variables.

For instance, the correlations between the richness of biocrust components were confounded by environmental variables. According to the bivariate correlation analysis, RMo was negatively correlated with RCA (*r* = –0.59), while this correlation was weak and nonsignificant in the SEM. This spurious correlation occurs because water availability has a strong influence on the richness of both biocrust components.

In addition, the environmental variables also confounded each other. For example, the variables in MF3 (pH, Ca, and SS) had negative correlations with the richness of mosses, as shown in Figures 4, 6, while MF3 showed a positive influence on RMo from SEM (Figure 5). These contrasting results come from that the correlations between MF1 or MF2 and MF3 are opposite to the influence of MF1 or MF2 on RMo. Therefore, these negative confounding influences will mask the positive correlation between MF3 and RMo, resulting in a spurious negative correlation. This contrasting result highlights the important impact of confounding influences, especially the major driving variables, on minor influences (Shipley, 2016). Besides, except for soil texture, we found no direct influence of MF1 and MF3 on the RLi from SEM (Figure 5). Luttge et al. (2011) discovered that excessive moisture is harmful to lichens because the desiccation–rehydration cycle damages the thalli of lichens. This evidence suggests a potential relationship between water and the richness of lichens, which is not supported by our findings. Our results also challenge the well–recognized association between gypsiferous soil and the high richness of lichens (Martínez et al., 2006; Ochoa-Hueso et al., 2011). We believe that previous studies conventionally used the canonical ordination method to study environmental drivers, but this approach cannot eliminate the confounding correlation between soil texture and soil salinity and sodicity.

## 5. Conclusion

The patterns of biocrust richness were determined across the arid and semiarid desert regions of China, and the main environmental indicators were examined. Latent variables related to water availability, soil texture, and soil salinity and sodicity representing the main environmental variations, drive the diversity patterns of biocrusts at the intracontinental scale. In specific, water availability has a positive influence on the richness of mosses and a negative influence on the richness of cyanobacteria–algae. Fine particle in soil texture has a negative influence on the richness of mosses and a positive influence on the richness of lichens. Soil salinity and sodicity have positive influences on the richness of mosses. By comparing the results of RDA and SEM fitting, we found that the correlations between the richness of biocrust components were confounded by environmental variables and the environmental variables also confounded with each other, which indicates that conventional RDA or canonical analysis may be strongly affected by confounding effects. Therefore, we suggest that any ecological inference revealed by the canonical ordination method should be taken with caution, and we argue that path analysis could be a good solution to eliminate such confounding effects.

## Data Availability Statement

The original contributions presented in the study are included in the article/Supplementary Material, further inquiries can be directed to the corresponding author.

## Author Contributions

JS: conceptualization, formal analysis, visualization, and writing. XL: methodology, project administration, and funding acquisition. All authors listed have made a substantial, direct, and intellectual contribution to the work and approved it for publication.

## Funding

This study was supported by the National Natural Science Foundation of China (Grant nos. 41621001 and 32061123006).

## Conflict of Interest

The authors declare that the research was conducted in the absence of any commercial or financial relationships that could be construed as a potential conflict of interest.

## Publisher's Note

All claims expressed in this article are solely those of the authors and do not necessarily represent those of their affiliated organizations, or those of the publisher, the editors and the reviewers. Any product that may be evaluated in this article, or claim that may be made by its manufacturer, is not guaranteed or endorsed by the publisher.

## Abbreviations

RDA: Redundancy analysis
SEM: Structural equation model
Silt: Silt content
Clay: Clay content
pH: Soil pH
SS: Soil soluble salt
Ca: *CaCO*_3_ accumulation
SOM: Soil organic matter
TN: Total nitrogen content
TP: Total phosphorus content
TK: Total potassium content
TSW: Topsoil water content
cvP: Perennial plants cover
BM: Biomass of biocrusts
RMo: Richness of mosses
RLi: Richness of lichens
RCA: Richness of cyanobacteria-algae.
