# Supplementary material for: Water Availability, Soil Characteristics, and Confounding Effects on the Patterns of Biocrust Diversity in the Desert Regions of Northern China
Source: Front Plant Sci. 2022 May 26;13:835668. doi: 10.3389/fpls.2022.835668 (PMC9199854; doi:10.3389/fpls.2022.835668)
Supplement: Supplementary file 1 [file Table_1.docx]

Appendix A: Overview of study sites

**Table A1** Climatic regime and vegetation composition of the study sites

| Study site | Horqin Desert  (Horqin) | western Loess Plateau  (Loess) | Mu Us Desert  (Mu Us) | Tengger-Alxa Desert  (Tengger) | Guerbantunggut Desert  (Guerban) | Qaidam Desert  (Qaidam) |
| --- | --- | --- | --- | --- | --- | --- |
| Area (×10^4^ km^2^) | 10.56 | >10 | 3.2 | 4.27 | 4.88 | 3.49 |
| Annual precipitation  (mm) | 450 | 430 | 335 | 186 | 110 | 80 |
| Months during which most precipitation fall | May- Sept. | May- Sept. | May- Sept. | May- Sept. | Jan.- March. | May- Sept. |
| Mean annual temperatures (^O^C) | 5.5 | 12.3 | 6.2 | 10.5 | 8.3 | 4.6 |
| Mean January temperature (^O^C) | -17.3 | -5.6 | -12.3 | -6.9 | -9.3 | -10.4 |
| Mean July  Temperature (^O^C) | 24.3 | 23.6 | 24.0 | 24.3 | 21.5 | 17.3 |
| Annual evaporation  (mm) | 2100 | 2300 | 2300 | 2600 | 2700 | 2900 |
| Vegetation composition  (dominant plants: frequency >5% in total investigative plots) | *Agorostis cristatum*  *Artemisia halodendron*  *Artemisia frigida*  *Atraphaxis brateata*  *Caragana microphylla*  *Cleistogenes squarrosa*  *Enneapogon borealis*  *Phragmies communis Pinus sylvestris var. Mongolica*  *Poa prateniss*  *Lespedeza davurica*  *Leymus secalinus*  *Salsola pestifer*  *Sonchus brachyotus*  *Stipa glareosa*  *Suaeda glauca*  *Setaria viridis* | *Agropyron cristatum*  *Allium polyrhizum*  *Artemisia gmelinii*  *Artemisia microphylla*  *Bothriochloa ischaemum*  *Caragana stenophylla*  *Ceratoides latens*  *Ephedra rhytidoperma*  *Eragrostis poaeoides*  *Kochia scoparia*  *Hippophae rhamnoides*  *Leymus secalinus*  *Plantago asiatica*  *Reaumuria soongarica*  *Salsola paserina*  *Stipa breviflora*  *Stipa bungeana*  *Poa annua* | *Artemisia ordosica Artemisia frigida*  *Astragalus melilotoides*  *Bassia dasyphylla*  *Caragana korshinskii*  *Caragana microphylla*  *Chenopodium aristatum*  *Corispermum mongolicum*  *Echinops gmelini*  *Euphorbia humifusa*  *Hedysarum laeve*  *Inula salsoloides*  *Lespedeza davurica*  *Oxytropis psammocharis Psammochloa villosa*  *Salsola ruthenica*  *Setaria viridis*  *Stipa breviflora*  *Scorzonera divaricata* | *Agriophyllum squarrosum*  *Allium mongolicum*  *Artemisia capillaris*  *Artemisia ordosica*  *Ammopiptantus mongolica*  *Bassia dasyphylla*  *Caragana korshinskii*  *Cleistogens songorica*  *Chloris virgata*  *Corispermum patelliforme*  *Echinops gmelini*  *Eragrostis poaeoides*  *Reaumuria soongarica*  *Stipa breviflora*  *Stipa gobica*  *Salsola paserina*  *Stipa glareosa* | *Artemisia arenaria*  *Aristida pennata*  *Agriophyllum squarrosum*  *Allium sp.*  *Bassia dasyphylla*  *Ceratocarpus arenarius*  *Corispermum lehmannianum*  *Echinops gmelinii*  *Eremurus anisopteris*  *Haloxylon persicum*  *Lappula rupestris*  *Salsola perstifercollina*  *Torularia torulosa* | *Aneurolepidium dasystachys*  *Artemisia capillaris*  *Bassia dasyphylla*  *Calligonum zaidamense*  *Carex przewalskii*  *Eragrostis poaeoides*  *Haloxylon ammodendrom*  *Leymus secalinus*  *Lyceum ruthenicum*  *Nitraria tangutorum*  *Reaumuria soongarica*  *Salsola ruthenica*  *Stipa glareosa*  *Stipa gobica*  *Phragmies communis*  *Poa annua* |
| Species of cyanobacteria and algae  (+++ indicates dominant species, ++ subdominant species, + rare species) | *Anabaena azotica* Ley (+++)  *Asterocapsa purpurea* (Jao) Chu (+)  *Characium angustum* A. Braun (+)  *Chlamydamonas* sp. (+)  *Chlorella vulgaris* Beij (++)  *Chloorococcum humicola* (Näg) Rab. (+)  *Microcystis densa* G. S. West (++)  *Nostoc* sp. (+++)  *Pediastrum boryanum* (Turp.) Menegh. (+)  *Synechocystis aquatilis* Sauv. (+)  *Synechocystis crassa* Woronichin (+) | *Anabaena azotic* Ley (+++)  *Brcteococcus* sp. (+)  *Microcoleus vaginatus* (Vauch.) Gm. (++)  *Moncilia* sp. (++)  *Myrmecia* sp. (++)  *Nostoc* sp. (+++)  *Hydrocoleus* sp. (+)  *Lyngbya digueti* Gom. (++)  *Oscillatoria nigra* Vauch. (+++)  *Phormidium mucicola* Naum. (++)  *Synechocystis aeruginosus* Näg (+)  *Tolypothrix* sp. (++) | *Anabaena azotica* Ley (++)  *Chroococcus minutus* (Kütz) Näg (+++).  *Diatoma vulgare* Bory. (++)  *Gloeocapsa arenaria* (Has.) Rebenh (+)  *G. aeruginosa* (Carm.) Kütz. (+)  *G. atrata* (Turp.) Kütz. (+)  *Gomphonema constrictum* Ehr. (+)  *Lyngbya digueti* Gom. (++)  *Microcoleus vaginatus* (Vauch.) Gm. (++)  *Penium cruciferum* (de Bary) Wittr. (++)  *Penium* spp. (+)  *Scytonema incrasstum* Jao (++)  *Scytonema stuposum* (Kütz.) Born. (++)  *Scytonema javanicum* (Kütz) Born. (++)  *Synechocystis crassa* Woronichin (++) | *Anabaena azotica* Ley. (++)  *Chlamydomonas* sp. (+)  *Chlorella vulgaris* Beij. (+)  *Chlorococcum humicola* (Naeg.) Rab. (++)  *Chroococcus epiphyticus* Jao (+)  *Cyambella* sp. (+)  *Desmococcus olivaceus* (Pers ex Ach) Laundon (+)  *Diatoma vulgare* var. Ovalis (Frick.) Hust. (++)  *Euglena* sp1. (++)  *Euglena* sp2. (++)  *Fragilaria intermedia* Grun. (+)  *Gloecapsa* sp. (+)  *Gomphonema constrictum* Ehr. (+)  *Hantzschia amphioxys* (Ehr.) Grun. (+)  *Lyngbya cryptovaginatus* Schk. (+++)  *Microcoleus vaginatus* (Vauch.) Gom. (+++)  *Navicula cryptocephala* Kütz (+)  *Nostoc flagelliforme* Born et Flah (++)  *Nostoc* sp. (+)  *Nostoc commune* (L.) Vauch. (+)  *Palmellococcus miniatus* (Kütz.) Chod. (++)  *Pinnularia borealis* Ehr. (++)  *Phormidium tenue* (Mengh.) Gom. (++)  *Synechocystis pevalekii* Ercegovic (+)  *Scytonema javanicum* Born. (++) | *Anabaena azotica* Ley (+++)  *Aphanocapsa delicatissima* W. et G.S.West (+)  *Calothrix* sp. (++)  *Chroococcus minutus* (Kütz) Näg. (++)  *Chroococcus turgidus* Näg (+++)  *Chroococcus westii* (W. West) Boy-Petersen (+)  *Clastidium* sp. (++)  *Gloeocapsa* sp. (+)  *Gomphosphaeria* sp. (++)  *Homoeothrix juliana* (Menegh.) Kiechn. (+)  *Hydrocoleus* sp. (+)  *Lyngbya martensiana* Meneghini (+)  *L. gracilis* Rabenh. (++)  *Microcoleus vaginatus* Gom. (+++)  *Microcoleus paludosus* (Kütz) Gom*.* (+++)  *Microcystis* sp. (+)  *Myxosarcina* sp. (+)  *Nodularia spumigena* Mertens (++)  *Nostoc* sp. (++)  *Oscillatoria agardhii* Gom. (++)  *O. cortiana* Menegh. (+)  *O. formosa* Bory (+)  *O. limosa* Ag. (++)  *O. tenuis* Ag. (++)  *Phormidium faveolarum* (Mont.) Gom.  *Porphyrosiphon* sp. (+)  *Raphidiopsis* sp. (+)  *Spirulina jenneri* (Stiz) Geiller (++)  *Symploca* sp. (+)  *Synechococcus* sp. (+)  *Synechocystis aeruginosus* Näg. (++)  *S. parvus* Mingula (++)  *Xenococcus lyngbyae* Jao (+++) | *Anabaena oscillarioides var. minor* Jao et Lee Kütz (++)  *A.* *variabilis* (++)  *Calothrix parietina* (Näg) Thuret (++)  *Chlorococcum humicola* (Naeg.) Rab. (+)  *Chroococcus epiphyticus* Jao (+++)  *Chroococcus* sp.(++)  *Gloeocapsa arenaria* (Has.) Rebenh(++)  *G. punctata* Näg (++)  *Homoethrix juliana* (Menegh.) Kirchn. (++)  *Hydrocoleus coccineus* Gom. (+)  *Lyngbya digueti* Gom. (+++)  *Microcolous tenerrimus* Gom. (++)  *M. vaginatus* Gom. (+++)  *Myxosarcina chroococcoides* Printz (+++)  *M. concinna* Printz (++)  *Navicula cryptocephala* Kütz (+)  *Nodularia harveyana* var. *sphaerocarpa* (Born. Et Flah.) Elenk. (+)  *Nostoc flagelliforme* Borm. (+++)  *N. humifusum* Carm.(+++)  *N. piscinale* Kütz (+)  *N. sphaericum* Vauch (+)  *N. spongaeforme* Ag. (+)  *Phormidium foveolarum* Gom. (+++)  *Porphyrosiphon* sp. (+)  *Schizothrix undulatus* Gom.(+++)  *Scytonema javanicum* (Kütz) Born. (+)  *Symploca* sp. (++)  *Synechocystis aeruginosus* Näg. (+)  *Synechocystis crassa* Woronichin (++)  *Tolypothrix* sp. (++) |
| Species of lichens  (+++ indicates dominant species, ++ subdominant species, + rare species) | *Acarospora schleicheri* (Ach.) Massal. (+)  *Fulgensia bracteata* (Hoffm.) Räsänen (+++)  *Glypholecia scabra* Müll. Arg. (+)  *Leptogium lichenoides* (l.) Zahlbr. (++)  *Peltogera leucophlebia* (Nyl.) Gyeln. (++) | *Collema coccophorum* Tuck. (+++)  *Collema tenax* (Sw.) Ach. Em. Degel (++)  *Diploschistes muscrum* (Scop.) Hoffm. (++)  *Endocarpon aridum* P. M. McCarthy (+++)  *E. pallidum* Ach. (+)  *E. pusillum* Hedw. (+)  *E. rogersii* P. M. McCarthy (+)  *E. rosettum* Amar Singh & Upreti (+)  *E. simplicatum* (Nyl.) Nyl. (++)  *Fulgensia bracteata* (Hoffm.) Räsänen (+++)  *Gyalidea asteriscus (Anzi) Aptroot & Lücking*  *ssp. Gracilispora J. Yang & J. C. Wei (+)*  *Heppia lutosa* (Ach.) Nyl. (++)  *Lecania mongolica* H. Magn (+)  *Placidium rufesens* (Ach.) A. Massal. (+)  *Psora decipiens* (Hedw.) Hoffm. (+++)  *Pyxine collina* (Ach.) Schrad. (+)  *Toninia sedifolia* (scop.) Timdal (++) | *Collema coccophorum* Web.(+++)  *C. tenax* (Sw.) Ach. (+++)  *Diploschistes muscorum* Sw.(++)  *Psora decipiens* (Ehrh.) Ach. (+++) | *Collema coccophorum* Tuck. (+++)  *Collema tenax* (Sw.) Ach. Em. Degel  *Diploschistes muscrum* (Scop.) Hoffm. (+++)  *Endocarpon aridum* P. M. McCarthy (++)  *E. crystallium* Wei & Yang (+)  *E. pallidum* Ach. (+)  *E. pusillum* Hedw. (+)  *E. rogersii* P. M. McCarthy (+)  *E. rosettum* Amar Singh & Upreti (+)  *E. simplicatum* (Nyl.) Nyl. (++)  *E. sinense* H. Magn. (+)  *Fulgensia bracteata* (Hoffm.) Räsänen (+++)  *Heppia lutosa* (Ach.) Nyl. (++)  *Lecania mongolica* H. Magn (+)  *Placidium rufesens* (Ach.) A. Massal. (+)  *Toninia sedifolia* (scop.) Timdal (++) | *Acarospora strigata* (Nyl.) Jatta. (++)  *Caloplaca songoricum* A. Abbas (++)  *Candeleriella aurella* (Hoffm.) Zahlbr. (+++)  *Catapyrenium* sp. (++)  *Collema tenax* (Sw.) Ach. Em. Degel (+++)  *Collema tenax* var. *corallinum* (Massal.)Degel (++)  *Dimelaena oreina* (Ach.) Nonnon (+)  *Diploschistes muscorum* (Scop.) R. Sant (+++)  *Fulgensia bracteata* (Hoffm.) Räsänen. (++)  *Lecanora argopholis* (Ach.) Ach. (++)  *Lecididea* sp. (+)  *Psora decipiens* (Ehrh.) Hoffm. (+++)  *Xanthoria elegans* (Link) Th. Fr. (++) | *Collema coccophorum* Web. (+++)  *C. Tenax* (SW.) Ach. (+)  *Diploschistes muscorum* (Scop.) R. Sant (++)  *Psora decipins* (Ehrh.) Ach. (++)  *Endocarpon* sp. (+)  *Toninia Sedifolia* (Scop.) Timdal (+) |
| Species of moss  (+++ indicates dominant species, ++ subdominant species, + rare species) | *Abietinella abietina* (Hedw.) Fleisch. (++)  *Amblystegium serpens* (Hedw.) B.S.G. var serpens (+)  *Anoectangium aestivum* (Hedw.) Mitt. (+)  *Anoectangium stracheyanum* Mitt. (++)  *Barbula unguiculata* Hedw. var. *unguiculata* (++)  *Brachythecium albicans* (Hedw.) B.S.G. (+)  *Brachythecium plumosum* (Hedw.) B.S.G. var nitdum  Tak. (+)  *Brachythecium perminusculum* C. Mull. (+)  *Bryoerythrophylle recurvirostre* Hedw.) Chen (+)  *Bryum arcticum* (R. Brown) B.S.G. (+)  *Bryum alpinum* Huds ex With. (+)  *Bryum argenteum* Hedw. (+++)  *Bryum dichotomum* Hedw. (+)  *Bryum kunzei* Hoppe et Hornsh (++)  *Bryum lonchocaulon* C. Müll. (+)  *Bryum pseudotriguetrum* (Hedw.) Gaertn. (+)  *Bryum pallescens* Schleich ex. Schwaegr. (++)  *Bryum thomsonii* Mitt. (++)  *Bryum uliginosum* (Brid.) B.S.G. (+++)  *Campylium chrysophyllus* J. Lange (+)  *C. hispidulum* (Brid.) Mitt. (+)  *Cratoneuron filicinum* (Hedw.) Spruc. (+)  *Crossidium crassinerve* (De Not.) Jur. (+)  *Desmatodon leucostoma* (R. Br.) Berggr. (+)  *Didymodon vinealis* (Brid.) Zand. (++)  *Drepanocladus aduncus* (Hedw.) Warnst. (++)  *Entodon caligiaosus* (Mitt.) Jaeg. (+)  *Entodon concinnus* (De Not.) Par. (++)  *Homomallium incurvatum* (Brid.) Loesk. (++)  *Lindbergia brachyptera* (Mitt.) Kindb. (+)  *Phascum cuspidatum* Schreb. Ex Hedw. (++)  *Pterygoneurum subsessile* (Brid.) Jour. (++)  *Pottia intermedia* (Turn.) Fuernr. (+)  *Pylaisiella brotheri* (Besch.) Iwats. et Nog. (+)  *Rauiella fujisana* (Par.) Reim. (+)  *Tortula mucronifolia* Schwaegr. (++)  *Weissia controversa* Hedw. (++) | *Aloina rigida* (Hedw.) Limpr. var rigida (++)  *Barbula fallax* Hedw. (++)  *Bryum algoricum* Sendt (+)  *Bryum argenteum* Hedw. (++)  *Bryum caespiticium* L. ex Hedw. (++)  *Bryum funkii* Schwaegtr (+)  *Crossidium crassinerve* (De Not.) Jur. (+)  *Didymodon constrictus* (Mitt.) Saito (+++)  *Didymodon nigrescens* (Mitt.) Saito (+++)  *Gymno stomum calcareum* Nee. et Honsch. (++)  *Plagiobryum zierii* (Hedw.) Lindb. (+)  *Pterygoneurum subsessile* (Brid.) Jour. (+) | *Aloina rigida* (Hedw.) var. *rigida* Limpr. (+)  *A. brevirostris* (Hook. & Grev.) Kindb. (++)  *A. crnifolia* Delogad. (+)  *A. obliquifolia* (C. Müll.) Broth. (+)  *Barbula indica* (Hook.) Spreng (+)  *B. unguiculata* var. *unguiculata* Hedw. (+)  *Bryoerythrophyllum recurvirostre* (Hedw.) Chen (+)  *Bryum argenteum* Hedw. (+++)  *B. caespiticium* L. ex Hedw. (++)  *Ceratodon purpureus* var. *purpureus* (Hedw.) Brid. (++)  *Crossidium chloronotos* (Brid.) Limpr. (+)  *C. crassinerve* (De Not.) Jur. (++)  *C. squamigerum* (Viv.) Jur. (++)  *Dicranella varia* (Hedw.) Schimp. (++)  *Didymodon* acutus (Brid.) Saito (+)  *D. constrictus* (Mitt.) Saito (++)  *D. icmadophyllus* (Schimp. ex C. Müll.) Saito (++)  *D. nigrescens* (Mitt.) Saito (+++)  *D. perobtusus* Broth. (++)  *D. rigidulus* Hedw. (+)  *Funaria hygrometrica* Hedw. (++)  *Gymnostomum calcareum* Nee. et Hornsch. (+)  *Hilpertia velenoviski* (Schiffn.) Zand. (++)  *Microbryum rectum* (With.) Zand. (++)  *Pterygoneurum subsessile* (Brid.) Jour. (+)  *P. ovatum* (Hedw.) Dix. (++)  *Tortula atrovirens* (Sm.) Lindb (+++)  *T. cernua* (Hueb.) Lindb.(++)  *T. desertorum* Broth (++)  *T. randii* (Kenn.) Zand. (+)  *Weissia controversa* Hedw. (+) | *Aloina brevirostris* Kindb.(+)  *A.obliquifolia* (C. Müll.) Broth. (+)  *A. rigida* (Hedw.) var. rigida Limpr. (+)  *Barbula ditrichoides* Broth. (+++)  *Bryoerythrophyllum recurvirostre* (+)  *Bryum argenteum* Hedw. (+++)  *Didymodon constrictus* (Mitt.) Saito (++)  *D. nigrescens* (Mitt.) Saito (+++)  *D. perobtusus* Broth. (++)  *D. tectorum* (C. Mull.) Saito (++)  *Pterygoneurum subsessile* (Brid.) Jur. (+)  *Tortula bidentata* Bai. X. L.(+++)  *T. desertorum* Broth. (++) | *Bryum argenteum* Hedw. (+++)  *B. capillare* L. ex Hedw. (++)  *Crassidium chloronotos* (+)  *Grimmia anodon* B. S. G. (+)  *G. pulvinata (Hedw.)* Sm. (+)  *Tortula desertorum* Broth. (++)  *T. muralis* Hedw. (+) | *Bryum argenteum* Hedw. (+++)  *B. caespiticium* Hedw. (+)  *Didymodon constrictus* (Mitt.) Saito (++)  *Pterygoneurum subsessile* (Brid.) Jour. (++) |
