# Supplementary material for: Water Availability, Soil Characteristics, and Confounding Effects on the Patterns of Biocrust Diversity in the Desert Regions of Northern China
Source: Front Plant Sci. 2022 May 26;13:835668. doi: 10.3389/fpls.2022.835668 (PMC9199854; doi:10.3389/fpls.2022.835668)

Appendix B: preliminary tests of different cluster methods

Jingyao Sun, Xinrong Li

3/8/2022

Clustering results are basically the same, except for the “single”, “median” and “centroid” method. But the “median” and “centroid” methods show reversed tree, thus, have been abandoned. Cluster method of ward.D, ward.D2, complete linkage, average-linkage and McQuitty suggested to cluster the environmental variables into 4 groups: Clay, Silt and TP; Ca, SS and pH; SOM, BM, cvP and TSW; and TK and TN. TK and TN had very weak relationships with each richness variable; thus, we excluded these variables from subsequent analyses. Codes and results are shown as follow:

library("magrittr")
library("pheatmap")
BSCdata <- read.csv("BSCdata.csv")
fc <- subset(BSCdata, select = c("TSW","BM","cvP",
 "pH","SS","Ca","silt","clay",
 "SOM","TN","TP","TK"))
sp <- subset(BSCdata, select =
 c("RCA","RLi","RMo"))
data_heat <- cor(sp$RCA, fc) %>%
 rbind(cor(sp$RLi, fc)) %>%
 rbind(cor(sp$RMo, fc))
row.names(data_heat) <- c("RCA","RLi","RMo")
colnames(data_heat) <- c("TSW","BM","cvP","pH","SS","Ca","Silt",
 "Clay","SOM","TN" ,"TP" ,"TK")

pheatmap(data_heat, cluster_row = FALSE,
 fontsize_number = 20,
 cutree_col = 4,treeheight_col=200,
 clustering_method= "ward.D",main="ward.D method")


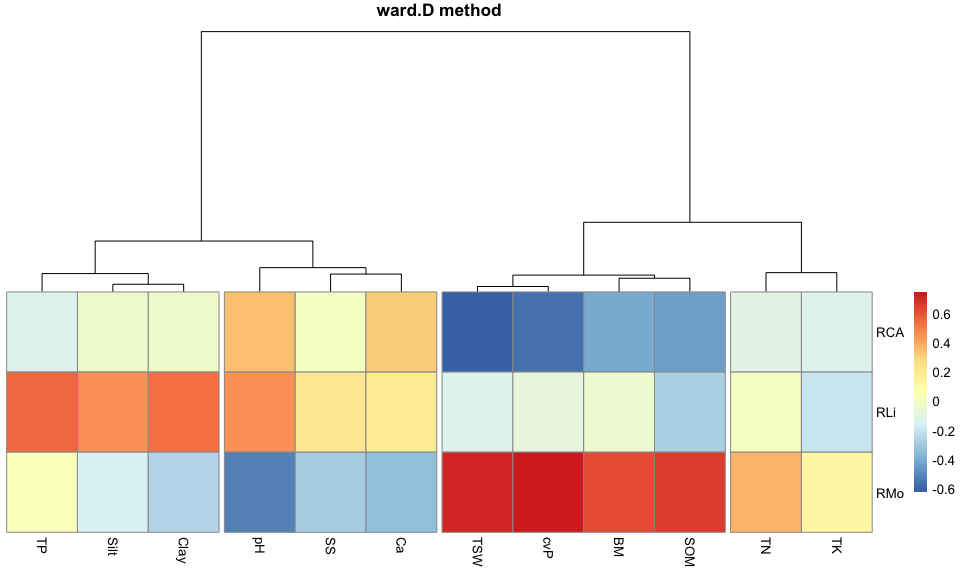


pheatmap(data_heat, cluster_row = FALSE,
 fontsize_number = 20,
 cutree_col = 4,treeheight_col=200,
 clustering_method= "ward.D2",main="ward.D2 method")


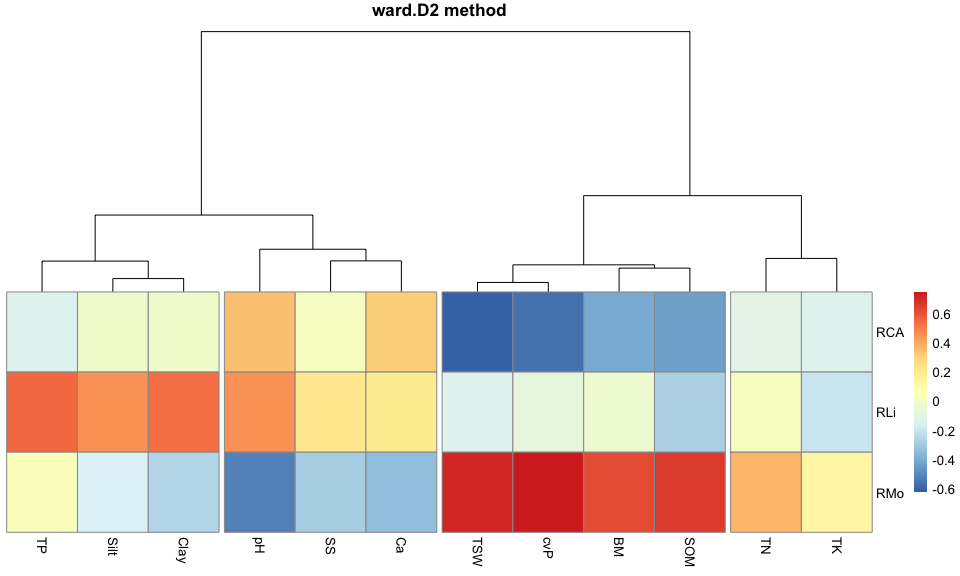


pheatmap(data_heat, cluster_row = FALSE,
 cutree_col = 4,treeheight_col=200,
 clustering_method= "single",main="single linkage")


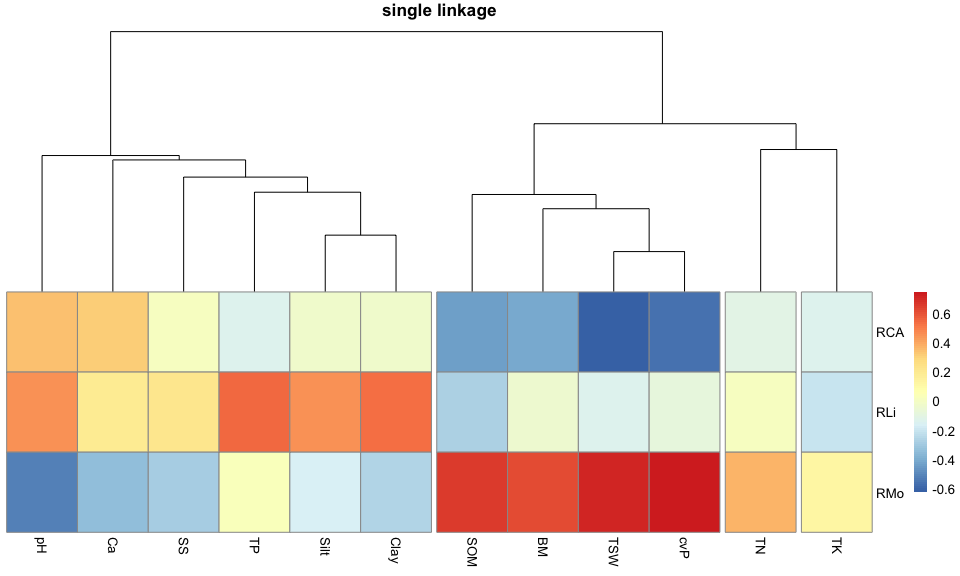


pheatmap(data_heat, cluster_row = FALSE,
 cutree_col = 4,treeheight_col=200,
 clustering_method= "complete",main="complete linkage")


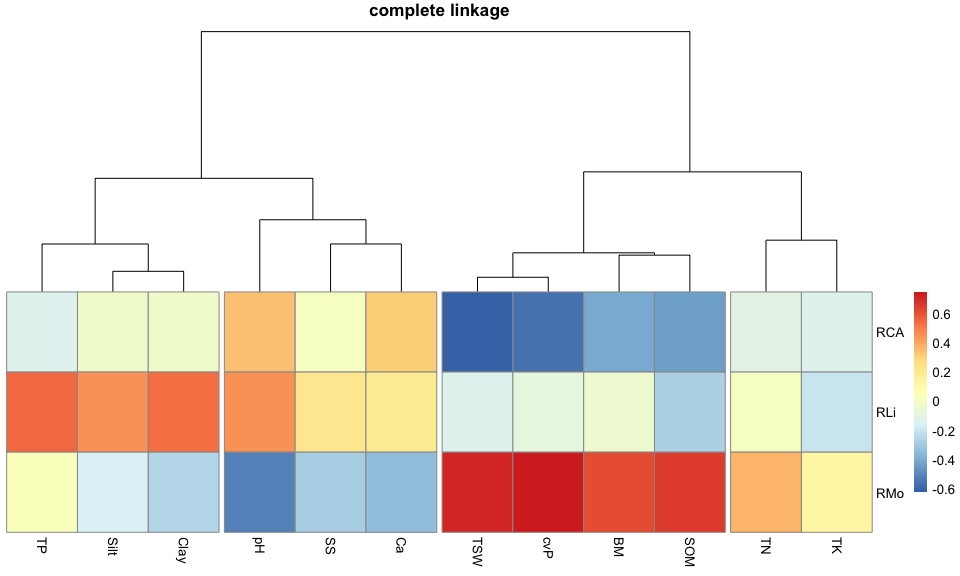


pheatmap(data_heat, cluster_row = FALSE,
 cutree_col = 4,treeheight_col=200,
 clustering_method= "average",main="average linkage")


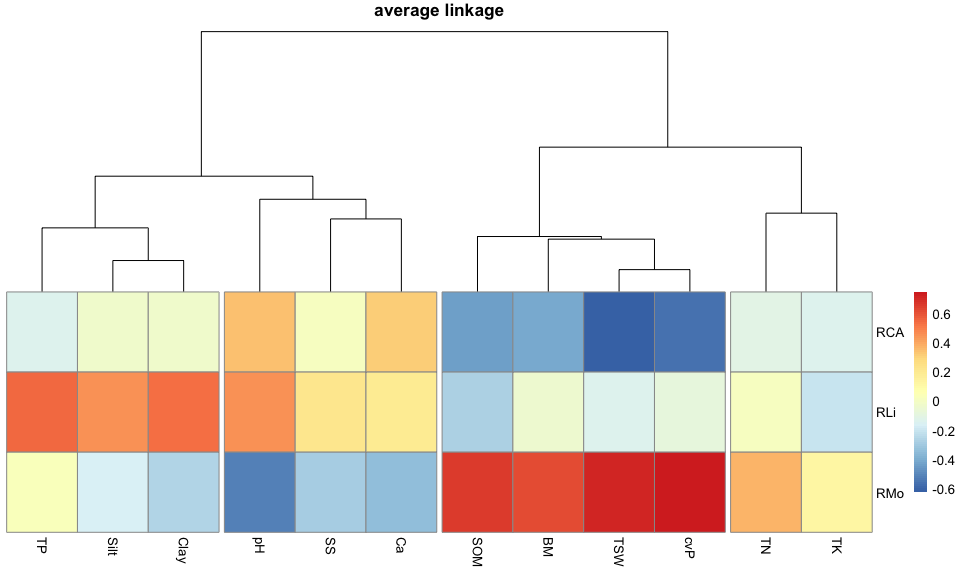


pheatmap(data_heat, cluster_row = FALSE,
 cutree_col = 4,treeheight_col=150,
 clustering_method= "mcquitty",main="MCQuitty method")


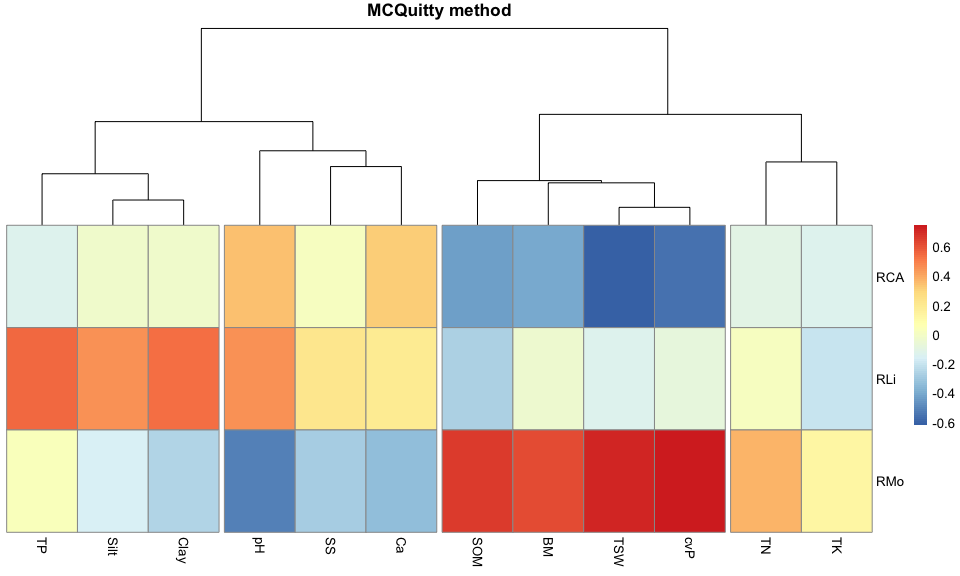


pheatmap(data_heat, cluster_row = FALSE,
 cutree_col = 4,treeheight_col=200,
 clustering_method= "median",main="median method")


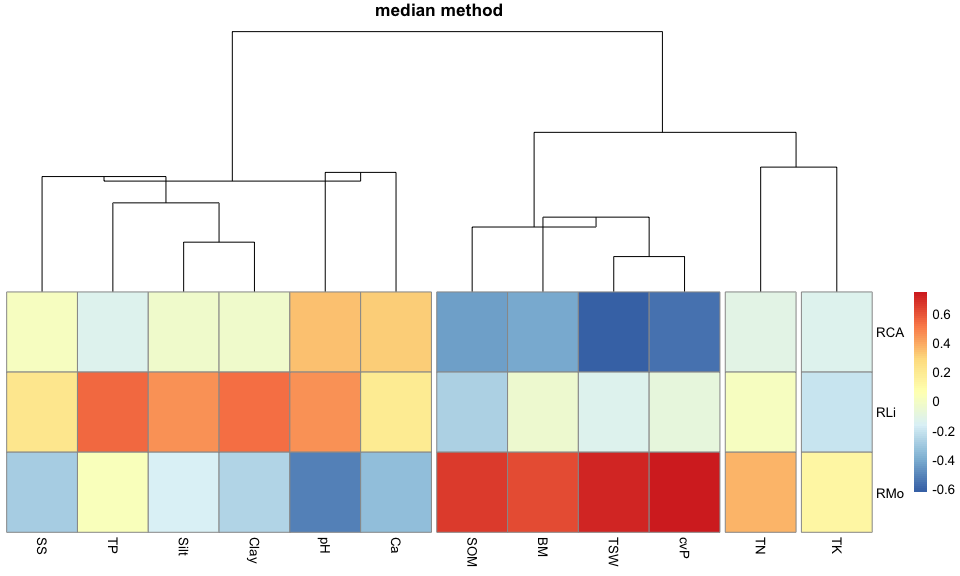


pheatmap(data_heat, cluster_row = FALSE,
 cutree_col = 4,treeheight_col=200,
 clustering_method= "centroid",main="centroid method")


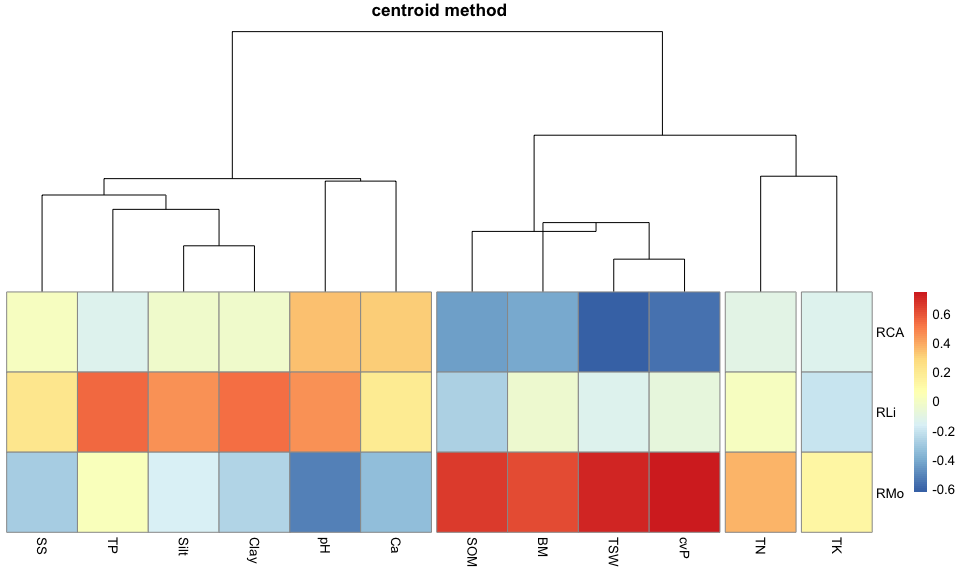

Supplement: Supplementary file 2 [file Table_2.DOCX]
